# MiR-155-5p Aggravated Astrocyte Activation and Glial Scarring in a Spinal Cord Injury Model by Inhibiting Ndfip1 Expression and PTEN Nuclear Translocation

**DOI:** 10.1007/s11064-023-03862-7

**Published:** 2023-02-07

**Authors:** Liming He, Qiang Chang, Yannan Zhang, Xiaoming Guan, Zhuo Ma, Xu Chen, Wenbo Liu, Yakun Li, Haoyu Feng

**Affiliations:** 1 Department of Orthopaedic Surgery, Shanxi Bethune Hospital, Shanxi Academy of Medical Sciences, Taiyuan, Shanxi China; 2 Department of Orthopaedic Surgery, Third Hospital of Shanxi Medical University, Taiyuan, Shanxi China; 3 Department of Orthopaedic Surgery, Tongji Shanxi Hospital, Taiyuan, Shanxi China

**Keywords:** Spinal cord injury, Astrocyte activation, Glial scarring, miR-155-5p, Ndfip1, PTEN

## Abstract

Central nervous injury and regeneration repair have always been a hot and difficult scientific questions in neuroscience, such as spinal cord injury (SCI) caused by a traffic accident, fall injury, and war. After SCI, astrocytes further migrate to the injured area and form dense glial scar through proliferation, which not only limits the infiltration of inflammatory cells but also affects axon regeneration. We aim to explore the effect and underlying mechanism of miR-155-5p overexpression promoted astrocyte activation and glial scarring in an SCI model. MiR-155-5p mimic (50 or 100 nm) was used to transfect CTX-TNA2 rat brain primary astrocyte cell line. MiR-155-5p antagonist and miR-155-5p agomir were performed to treat SCI rats. MiR-155-5p mimic dose-dependently promoted astrocyte proliferation, and inhibited cell apoptosis. MiR-155-5p overexpression inhibited nuclear PTEN expression by targeting Nedd4 family interacting protein 1 (Ndfip1). Ndfip1 overexpression reversed astrocyte activation which was induced by miR-155-5p mimic. Meanwhile, Ndfip1 overexpression abolished the inhibition effect of miR-155-5p mimic on PTEN nuclear translocation. In vivo, miR-155-5p silencing improved SCI rat locomotor function and promoted astrocyte activation and glial scar formation. And miR-155-5p overexpression showed the opposite results. MiR-155-5p aggravated astrocyte activation and glial scarring in a SCI model by targeting Ndfip1 expression and inhibiting PTEN nuclear translocation. These findings have ramifications for the development of miRNAs as SCI therapeutics.

## Introduction

Spinal cord injury (SCI) is a kind of destructive nerve injury, which is caused by trauma, disease, and other reasons, resulting in the injury stage and severe dysfunction of lower limbs, mostly in the elderly [1]. SCI directly acts on the nerves, causing damage to the nervous system and thus affecting the sensory, motor, and autonomic nerve functions of patients [2]. The extent and clinical manifestations of SCI depend on the site and nature of the primary injury [3]. At present, SCI has limited therapeutic effects and cannot effectively restore neurological functions, causing great physiological and psychological pressure on patients[4]. Therefore, the molecular basis research of neural system regeneration and repair is not only of great scientific significance but also of great social benefits.

Microglia activation, astrocyte proliferation and glial scar formation after SCI are the main factors preventing effective regeneration and growth of injured spinal cord axons [5, 6]. At present, the research on the glial scar in spinal cord injury has become a hot topic [7]. Glial scar is one of the important characters of SCI, which play a key role in the failure regeneration of axon after SCI [8]. Reactive gliosis is one of the important changes in chronic period. The development of the SCI which eventually leaves a huge lesion composed of large cysts that are totally walled off by a dense network of reactive astrocyte [8, 9]. Astrocytes comprise a large part of the glial scar and they have two roles to injuryed spinal cord. First, they seclude the injury site from healthy tissue, preventing a cascading wave of uncontrolled delayed injury. The second, they impeded the axonal regeneration [7, 10]. However, the molecular mechanism of regulation of glial scar formation remains unclear. Meanwhile, there are not appropriate methods to remove the glial scar in previous reports.

MicroRNA (miR) is a kind of single-stranded non-coding small RNA with a length of about 22–24 nucleotides [11, 12]. MicroRNA negatively regulates the function of the target protein by degrading the target gene mRNA or inhibiting its translation process[13]. Numerous studies have shown that miRNAs play an important role in the development of a variety of diseases, including SCI [14–16]. MiR-92b-3p promotes neurite growth and functional recovery via the PTEN/AKT pathway in acute SCI [17]. MiR-17 targeted PTEN and facilitated glial scar formation after spinal cord injuries via the PI3K/Akt/mTOR pathway [18]. MiR-21 regulated astrocytic reaction post-acute phase of SCI through modulating the TGF-β signaling pathway [19]. Particularly, the study confirmed that miR-155 knockdown could protect LPS-induced microglial BV2 inflammatory injury by regulating RACK1 expression and deactivating the MAPK/NF-κB and mTOR signaling pathway [20]. In a model of SCI, miR-155 deletion improved locomotor function at post injury times, which was associated with decreased accumulation of inflammatory macrophages [21]. However, the role of miR-155-5p on astrocyte activation and glial scar formation after SCI has not been reported. In this study, we explored how miR-155-5p participates in the astrocyte activation and scarring after SCI in vitro and in vivo. Our study provides a new treatment strategy for SCI therapy.

## Materials and Methods

### Cell Culture and Treatment

CTX-TNA2 rat brain primary astrocyte cell line (CRL-2006) was obtained from purchased from American Type Culture Collection (Manassas, VA, USA). The cells were cultured (37 °C, 5% CO_2_) in Dulbecco’s Modified Eagle’s Medium (DMEM; Gibco, Carlsbad, CA, USA) supplemented with 4.5 g/L glucose and 10% (v/v) and 10% heat-inactivated fetal bovine serum (FBS, Hyclone, South Logan, UT, USA). Transient transfection of miR-155-5p mimic/NC mimic (final concentration of 50 nM), or pcDNA-Ndfip1 (final concentration of 50 nM) was executed using Lipofectamine®2000 (Invitrogen, Grand Island, NY).

### Experimental Animals

Female Sprague-Dawley (SD) rats (weight, 200–220 g, 8–12 weeks old) were purchased from the Sichuan Dashuo Experimental Animal Co. Ltd. (license number: SCXK(chuan) 2020-034). The feeding environment was 23 ± 1 °C, relative humidity 55 ± 5%, and light/darkness for 12 h circulation. The rats are allowed to eat and drink freely. All experimental protocols were approved by the ethics review committee of Shanxi Bethune Hospital (No. SBQDL-2022-004). All methods are reported by ARRIVE guidelines (https://arriveguidelines.org/) for the reporting of animal experiments.

### Cell Proliferation Assay

According to the manufacturer’s instructions, cell proliferation of astrocytes was measured by the cell counting kit-8 (CCK-8, Dojindo, Japan) assay. In short, a total of 2.0 × 10^3^ cells/well were seeded into 96-well plates, with 10 µL of CCK-8 solution added to each well containing 10 µL of DMEM medium and incubated at 37 °C under 5% CO_2_ for 3 h. After 48 h incubation, absorbance at 450 nm was recorded by a microplate reader (Spectra Max Plus 384, Molecular Devices, Sunnyvale, CA, USA).

### Wound Healing Assay

Astrocytes were seeded into 6-well plates for 24 h to reach 80–90% confluence. The well was scratched with a 10 µl pipette to create a single wound in the center of the cell monolayer. Subsequently, cells were cultured in a serum-free MEM medium for 24 h at 37 °C. The wound healing distance was measured under an Olympus X51 inverted microscope (Olympus, Tokyo, Japan). Relative wound recovery (%) = (initial wound width - wound width at 24 h)/initial wound width ×100.

### Flow Cytometry Assay

The apoptosis of astrocytes was analyzed using an Annexin V-according to the manufacturer’s protocol. Briefly, the cells were washed with PBS (Invitrogen, Carlsbad, CA, USA), and adjusted the cell concentration to 1.0 × 10^6^ cells/mL. The cells were subsequently suspended in a 150 µl buffering solution. Subsequently, the cells were stained with 10 µg/ml Annexin V-FITC and 5 µl PI at 4˚C for 20 min in darkness. Apoptotic cells were then analyzed by a BD FACSCelesta™ Flow Cytometer (Becton, Dickinson, and Company).

### Dual-Luciferase Reporter Assay

Ndfip1 3’UTR (wt/mut) were combined into a psiCHECK-2 vector (Promega, Madison, USA). Afterward, the luciferase reporter gene plasmid and miR-155-5p mimic or NC mimic were co-transfected into 293T cells (4 × 10^4^ cells/well) using the Lipofectamine 3000 (Invitrogen, Carlsbad, CA, USA). After transfection for 24 h, the relative luciferase activity was detected by a luciferase reporter assay system.

### RNA Extraction and Reverse-Transcription Quantitative PCR (RT-qPCR)

TRIzol (Invitrogen, Carslbad, CA) reagent was used to extract total RNA from CDs according to the manufacturer’s protocol. RT-qPCR was performed by using the SYBR Premix Ex TaqTMII kit (Takara, Tokyo, Japan). TRT-qPCR was performed with a program of 5 min at 95 °C and then 45 cycles at 95 °C (30 s), 95 °C (5 s), 55 °C (30 s), and 72 °C (30 s). The cycle threshold (CT) values of the samples were analyzed using Thermo Scientific PikoReal software 2.1 (Thermo Fisher Scientific, Waltham, MA, USA). The 2^-ΔΔCT^ method (ΔΔCT = ΔCT_treatment_- ΔCT_control_ and ΔCT = Ct _target_ - Ct _reference_.) was used to calculate the relative expression. Each candidate gene was internally normalized against β-actin.

### Establishment of the SD Rat Model of SCI

SD rats were randomly divided into six groups: Sham (n = 8), SCI (n = 7), SCI + NC antagonist (n = 6), SCI + miR-155-5p antagonist(n = 8), SCI + NC agomir (n = 7), and SCI + miR-155-5p agomir group (n = 6). The surgical procedure was carried out as previously described [22, 23]. Briefly, SD rats received intraperitoneal anesthesia with 1% pentobarbital (30 mg/kg) before spinal cord injury. A byand laminectomy was performed at the T9-T11 spinal vertebrae to expose the spinal cord. Then, a 2 mm segment of the spinal cord was cut using microsurgical scissors and removed. The rats in the sham group underwent the same surgical procedure but without spinal cord injury. After SCI, the bladder of each rat was manually emptied thrice per day until bladder function returned. Penicillin (200,000 IU/d, intramuscular injection) was administrated to prevent bacterial infection. 30 min after spinal cord injury, rats in the SCI + NC antagonist, SCI + miR-155-5p antagonist, SCI + NC agomir, and SCI + miR-155-5p agomir group were treated with NC antagonist, miR-155-5p antagonist, NC agomir, or miR-155-5p agomir by spinal intrathecal injection, respectively (100 µmol/L, 50 µl/d, 3 consecutive days). Rats in the sham and SCI groups were injected with the equivalent volume of normal saline for 3 days. The motor function of the lower limbs in all rats was evaluated using the BBB score after SCI operation on days 0, 3, 7, and 14 days. Following completion of the trial, rats were euthanized using an overdose of pentobarbital sodium on day 14. In each group, six rats were used for behavioral testing, histological study, and western blot analysis.

### Basso-Beattie-Bresnahan (BBB) Scores

The motor function of the rats was assessed at the corresponding time points using the BBB rating scale, blind to the experimental condition. In short, the BBB scoring criteria were divided into 21 scores (ranging from 0 to 21). The movement of the hip, knee, and ankle joints, as well as the recovery of walking gait and coordinated movement ability, were observed.

### Western Blot Analysis

Spinal cord tissues and astrocytes were lysed in RIPA buffer (Signaling Technology, Inc., Danvers, MA, USA) to collect total proteins. Cytosolic and nucleus protein of astrocytes was extracted with Nuclear Extraction Kit (ab113474, abcam, Cambridge, UK). The protein content was quantitated using a BCA kit (Beyotime, Shanghai, China). Total protein (30 µg/sample) was separated via 10% SDS-PAGE and transferred to nitrocellulose membranes (Pall Life Sciences, New York, USA). The membranes were blocked with 5% nonfat dry milk for 1 h to prevent nonspecific binding of antibodies. After being probed with primary antibodies overnight at 4 °C, the membranes were washed and incubated with appropriate peroxidase-conjugated secondary antibodies. The corresponding protein primary antibodies were as follows: GAP43 (No. ab75810, Abcam, Cambridge, MA, USA; 1:1000), NF-200 (No. sc-32,729, Santa Cruz Biotechnology, Santa Cruz, CA, Inc.; 1:300), Ndfip1 (No. 236,892, Abcam, Cambridge, MA, USA; 1:500), PTEN (No. 267,787, Abcam, Cambridge, MA, USA;1:1000), and β-actin (No. ab8227, Abcam, Cambridge, MA, USA; 1:1000). Finally, the bands were visualized using the ECL system (Thermo Scientific, Rockford, IL, USA).

### Immunofluorescence (IF) Stain

Spinal cord cryosections and astrocytes coverslips were performed as previously described [24]. Then, the samples were incubated with Th1 antibody Ndfip1 (No. 236892, Abcam, Cambridge, MA, USA; 1:50) or PTEN (No.A19101, Abclonal, Wuhan, China; 1:50) at 4°C overnight. FITC goat anti-rabbit IgG was used as a secondary antibody. 4’,6-Diamidino-2-Phenylindole (DAPI, Sigma-Aldrich, USA) was added dropwise into the sections for 5 min. The staining was observed under a fluorescence microscope Olyvia (Olympus, Tokyo, Japan) at 400× magnifications. Image processing was conducted with Image J (National Institutes of Health, Bethesda, MA, USA).

### Statistical Analysis

A priori power analysis to obtain statistical significance (p = 0.05, power 80%) resulted in an n of 3 or 6 for each group after body-size adjustment. The data were represented as means ± standard deviation (SD). Statistical analysis was performed with the SPSS software (version 19.0, SPSS Inc., Chicago, IL, USA). One-way analysis of variance (ANOVA) with Tukey’s post hoc test was used for statistical analysis. Differences with a P < 0.05 were considered to indicate statistically significant.

## Results

### MiR-155-5p Overexpression Promoted Astrocyte Activation In Vitro

**Fig. 1:**
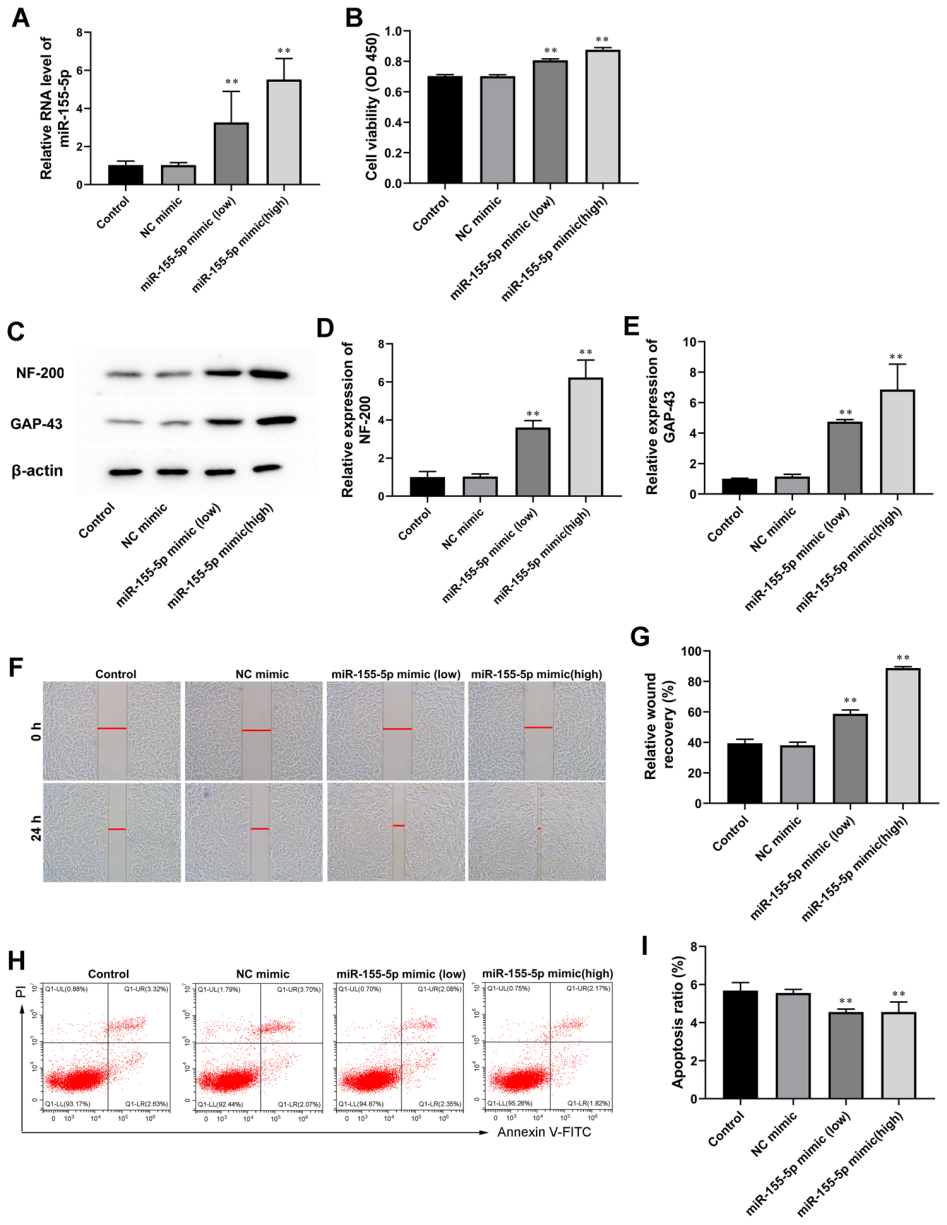
MiR-155-5p overexpression promoted astrocyte activation in vitro. MiR-155-5p mimic (50 nM) or miR-155-5p mimic (100 nM) was used to transfect astrocytes for 24 h. **A** MiR-155-5p expression levels were evaluated by RT-qPCR. **B** The effect of miR-155-5p mimic on the proliferation of astrocytes was detected by the CCK-8 test. **C**-**E** Western blot was used to assay determined NF-200 and GAP-43 expression. β-actin is an internal reference. **F** and **G** The effect of miR-155-5p mimic on astrocyte invasion was detected by Wound-healing assay. **H** and **I** Cell apoptosis was checked by flow cytometry. Values are means ± SD. *P < 0.05 vs. NC mimic, **P < 0.01 NC mimic

Firstly, we explored the effect of miR-155-5p mimic on astrocyte proliferation, invasion, and apoptosis. CTX-TNA2 cells were transfected with miR-155-5p mimic ( 50 nM or 100 nM) or or control miRNA mimic (NC mimic). The expression levels of miR-155-5p were significantly increased by miR-155-5p mimic (50 nM or 100 nM) in CTX-TNA2 cells (Fig. 1A). As shown in Fig. 1B, in the presence of miR-155-5p mimic, astrocytes exhibited increased proliferation, and a high concentration (100 nM) had a stronger effect than a low concentration (50 nM). Meanwhile, miR-155-5p overexpression astrocytes with miR-155-5p mimic showed a higher expression of NF-200 and GAP43 (Fig. 1C–E). MiR-155-5p overexpression concentration-dependently promoted astrocyte invasion (Fig. 1F and G) and inhibited astrocyte apoptosis (Fig. 1H and I).

### MiR-155-5p Overexpression Inhibited Nuclear PTEN Expression by Targeting Ndfip1

**Fig. 2:**
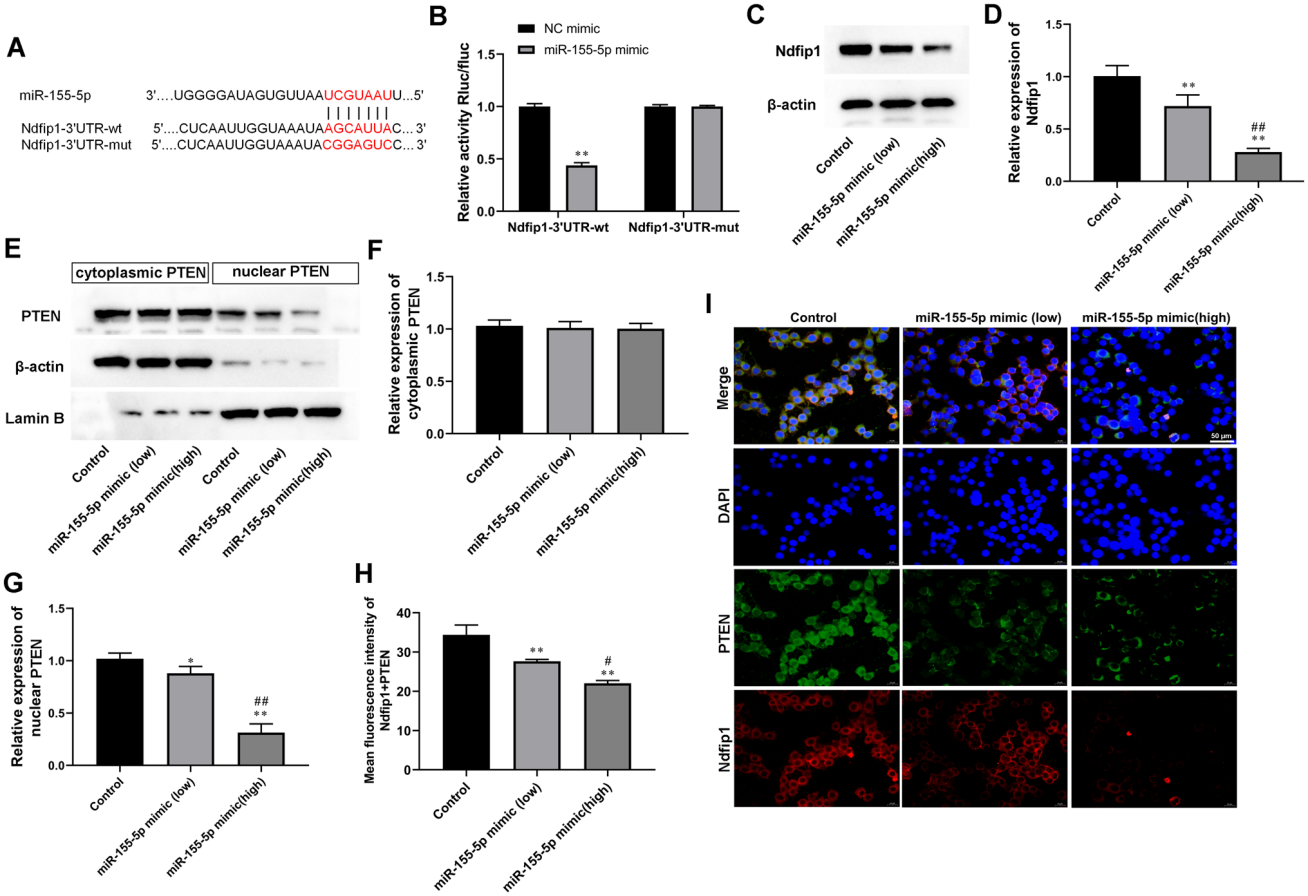
MiR-155-5p overexpression inhibited nuclear PTEN expression by targeting Ndfip1. MiR-155-5p mimic (50 nM) or miR-155-5p mimic (100 nM) was used to transfect astrocytes for 24 h. **A** The predicted miR-155-5p binding site in the 3’UTR sequences of Ndfip1. **B** MiR-155-5p negatively regulated the luciferase activity of Ndfip1-3’UTR-wt, rather than Ndfip1-3’UTR-wt in 293T cells. **C** and **D** Relative Ndfip1 expression was compared by Western blot analysis. β-actin is an internal reference. **E**–**G** The expression of PTEN in the cytoplasm and nucleus of was astrocytes compared by Western blot analysis. β-actin and Lamin B served as cytoplasmic and nuclear internal references, respectively. Values are means ± SD. **H** and **I** Immunofluorescence staining for PTEN and Ndfip1 in astrocytes. Scale bars = 50 μm. *P < 0.05 vs. control, **P < 0.01 vs. control, ^#^*P* < 0.05 vs. miR-155-5p mimic (low), ^##^*P* < 0.01 vs. miR-155-5p mimic (low)

To investigate the targeted genes of miR-155-5p, bioinformatics predictions (StarBase and TargetScan) and luciferase assay were used. we found that miR-155-5p might interact with Ndfip1 3’ UTR (Fig. 2A). Luciferase reporter assay showed that miR-155-5p decreased the luciferase activity of Ndfip1 luciferase reporters obviously (Fig. 2B). We then overexpressed miR-155-5p through miR-155-5p mimic transfection in astrocytes. As shown in Fig. 2C and D, miR-155-5p mimic transfection decreased the expression of Ndfip1 in astrocytes. Moreover, miR-155-5p overexpression concentration-dependently decreased nuclear PTEN expression (Fig. 2E and G). This result was further validated by IF staining (Fig. 2 H and 2I).

### MiR-155-5p Overexpression Stimulated Astrocyte Activation by Inhibiting Ndfip1 Overexpression

**Fig. 3:**
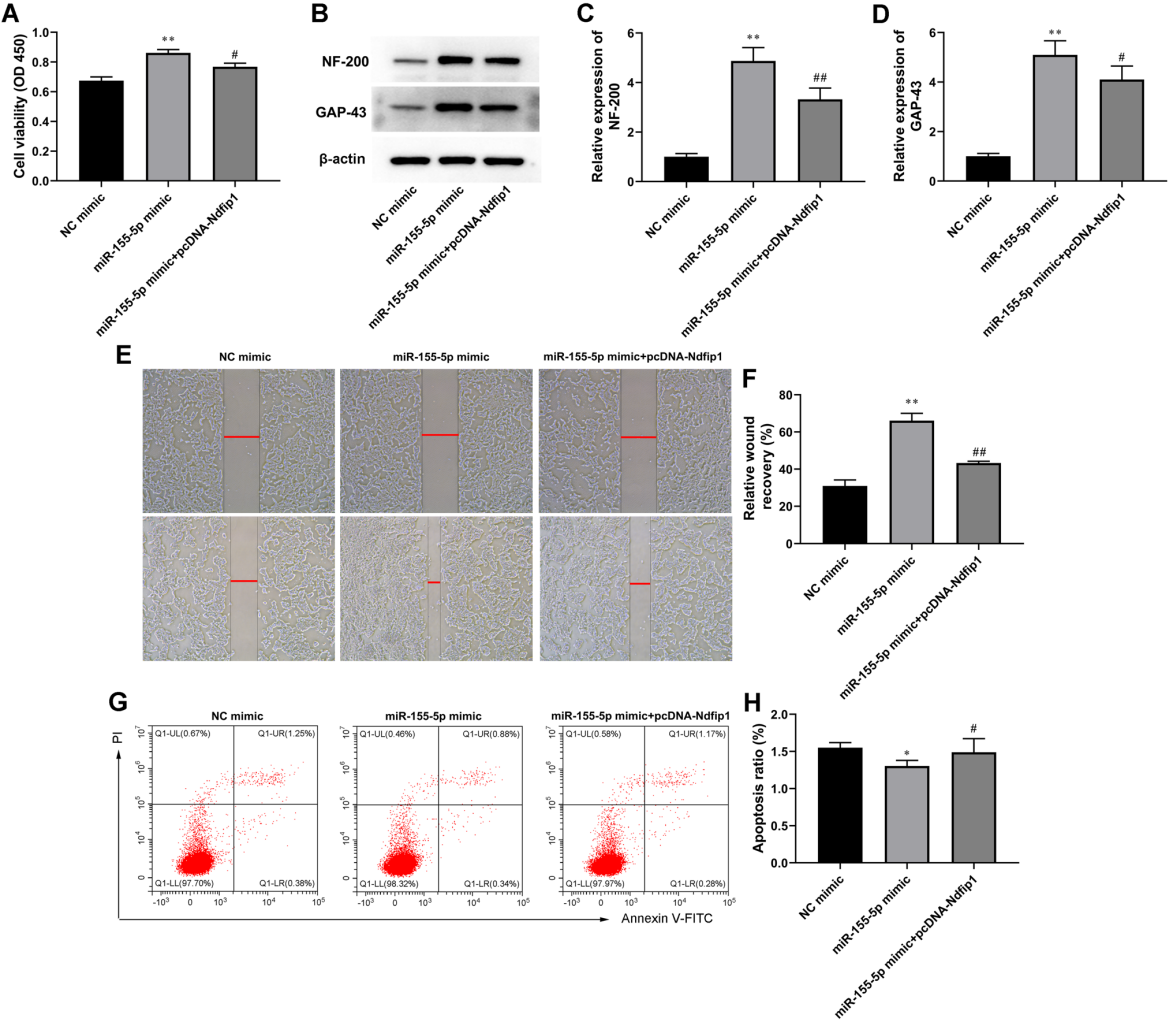
MiR-155-5p overexpression stimulated astrocyte activation by inhibiting Ndfip1 overexpression. NC mimic (100 nM), MiR-155-5p mimic (100 nM) or/and pcDNA- Ndfip1 were used to transfect astrocytes for 24 h. **A** Cell proliferation of astrocytes was tested by CCK-8 assay. **B**-**D** Western blot was used to assay determined NF-200 and GAP-43 expression. β-actin is an internal reference. **E** and **F** The effect of miR-155-5p mimic on astrocyte invasion was detected by Wound-healing assay. **G** and **H **Cell apoptosis was checked by flow cytometry. Values are means ± SD. *P < 0.05 vs. NC mimic, **P < 0.01 vs. NC mimic, ^#^*P* < 0.05 vs. miR-155-5p mimic, ^##^*P* < 0.01 vs. miR-155-5p mimic

The reporter plasmids were cotransfected with pcDNA expressing Ndfip1 into astrocytes. As shown in Fig. 3A, Ndfip1 overexpression reversed the increases in cell proliferation induced by the miR-155-5p mimic. In addition, the expression of NF-200 and GAP43 was reduced compared with miR-155-5p mimic-transfected astrocytes (Fig. 3B, C).

Intriguingly, we also observed that the increases in astrocyte invasion induced by the miR-155-5p mimic were dramatically blocked by pcDNA-Ndfip1 cotransfection (Fig. 3E, F). Further, we observed that the apoptosis of astrocytes was significantly decreased by miR-155-5p mimic, which was increased by pcDNA-Ndfip1 cotransfection (Fig. 3G, H).

### Ndfip1 Overexpression Abolished the Inhibition Effect of miR-155-5p Mimic on PTEN Nuclear Translocation

**Fig. 4:**
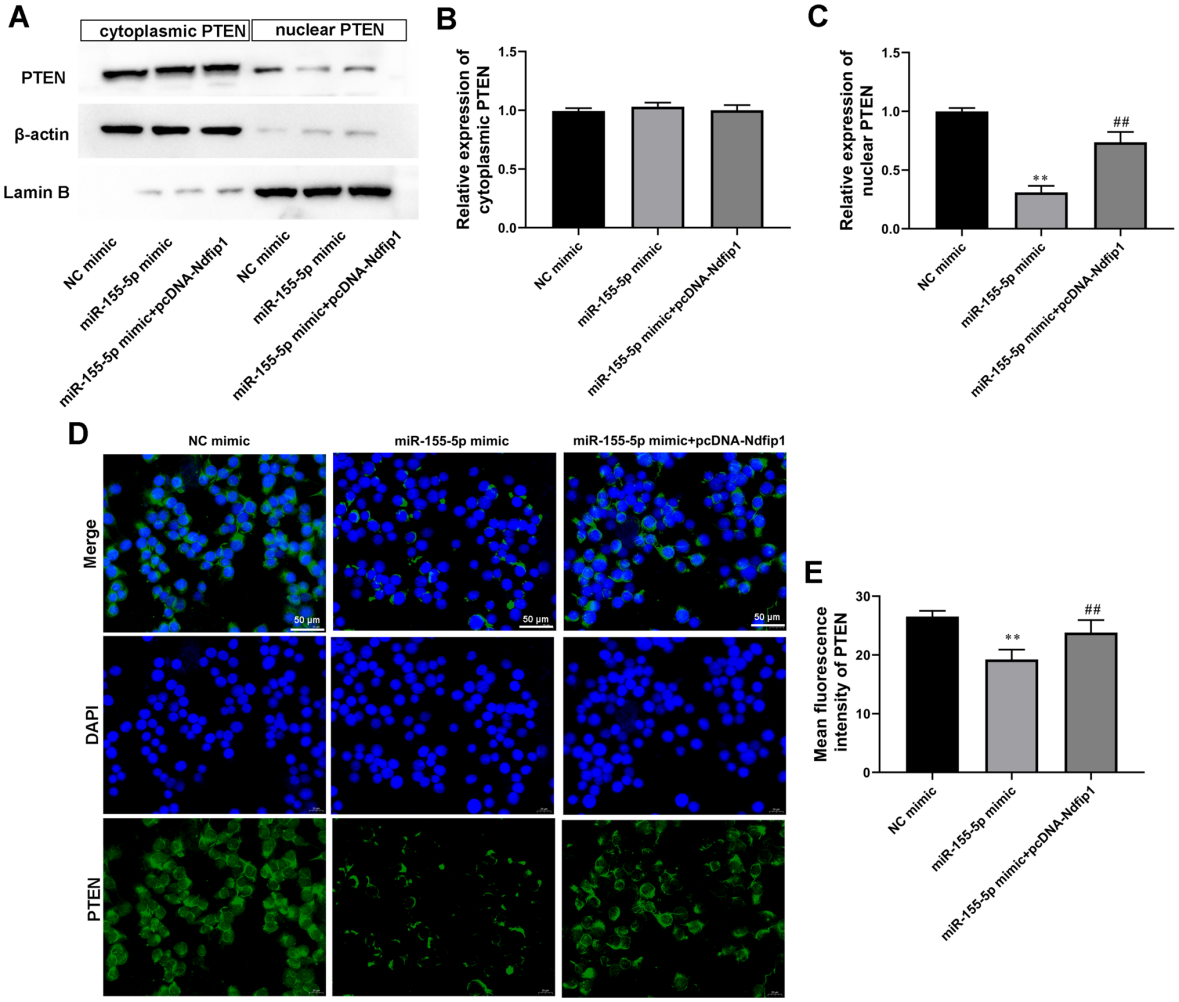
Ndfip1 overexpression abolished the inhibition effect of miR-155-5p mimic on PTEN nuclear translocation. **A** and **C** The expression of PTEN in the cytoplasm and nucleus of was astrocytes compared by Western blot analysis. β-actin and Lamin **B** served as cytoplasmic and nuclear internal references, respectively. **D** and **E** PTEN immunofluorescence stain in astrocytes. Scale bars = 50 μm. Values are means ± SD. **P < 0.01 vs. NC mimic, ^##^*P* < 0.01 vs. miR-155-5p mimic

Western blot analysis demonstrated that overexpression of Ndfip1 promoted the expression of nuclear PTEN in astrocytes compared with miR-155-5p mimic-transfected group (Fig. 4A–C). IF analysis also confirmed these results (Fig. 4D and E). Therefore, it was concluded that the overexpression of miR-155-5p suppressed PTEN nuclear translocation in astrocytes by targeting Ndfip1.

### MiR-155-5p Regulated Ndfip1 Expression and PTEN Nuclear Translocation In Vivo

**Fig. 5:**
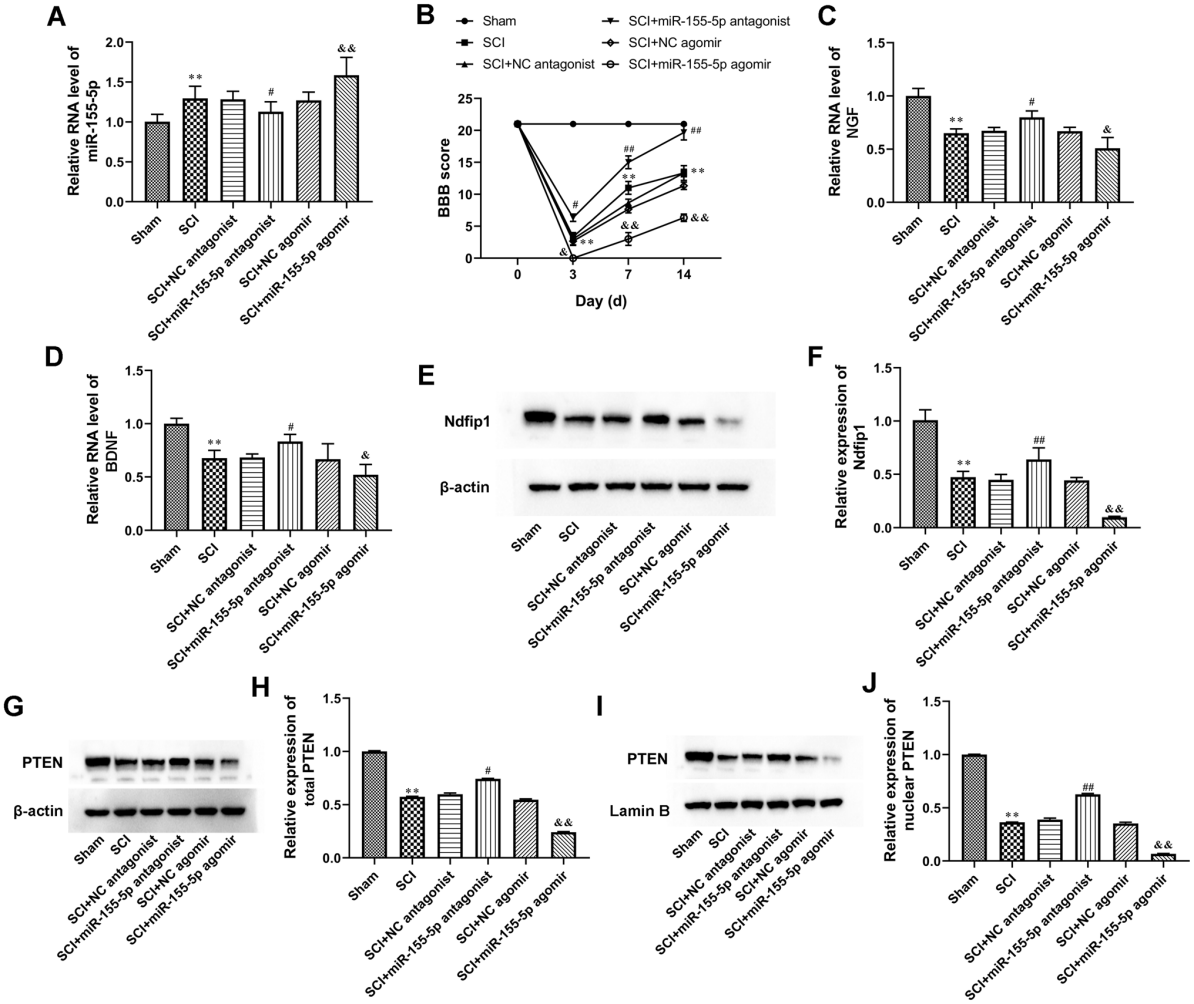
MiR-155-5p regulated Ndfip1expression and PTEN nuclear translocation in vivo. **A** The levels of miR-155-5p in the spinal cord were tested by RT-qPCR. **B** Basso-Beattie-Bresnahan (BBB) scale scores over time after spinal cord injury (SCI). **C** and **D** The levels of NGF and BDNF in the spinal cord were analyzed by RT-qPCR. **E**–**J** Western blot was used to assay determined the expression of Ndfip1 and PTEN in the spinal cord. β-actin and Lamin B served as cytoplasmic and nuclear internal references, respectively. **P < 0.01 vs. Sham, ^#^*P* < 0.05 vs. SCI + NC antagonist, ^&^*P* < 0.05 vs. SCI + NC agomir, ^&&^*P* < 0.01 vs. SCI + NC agomir

**Fig. 6:**
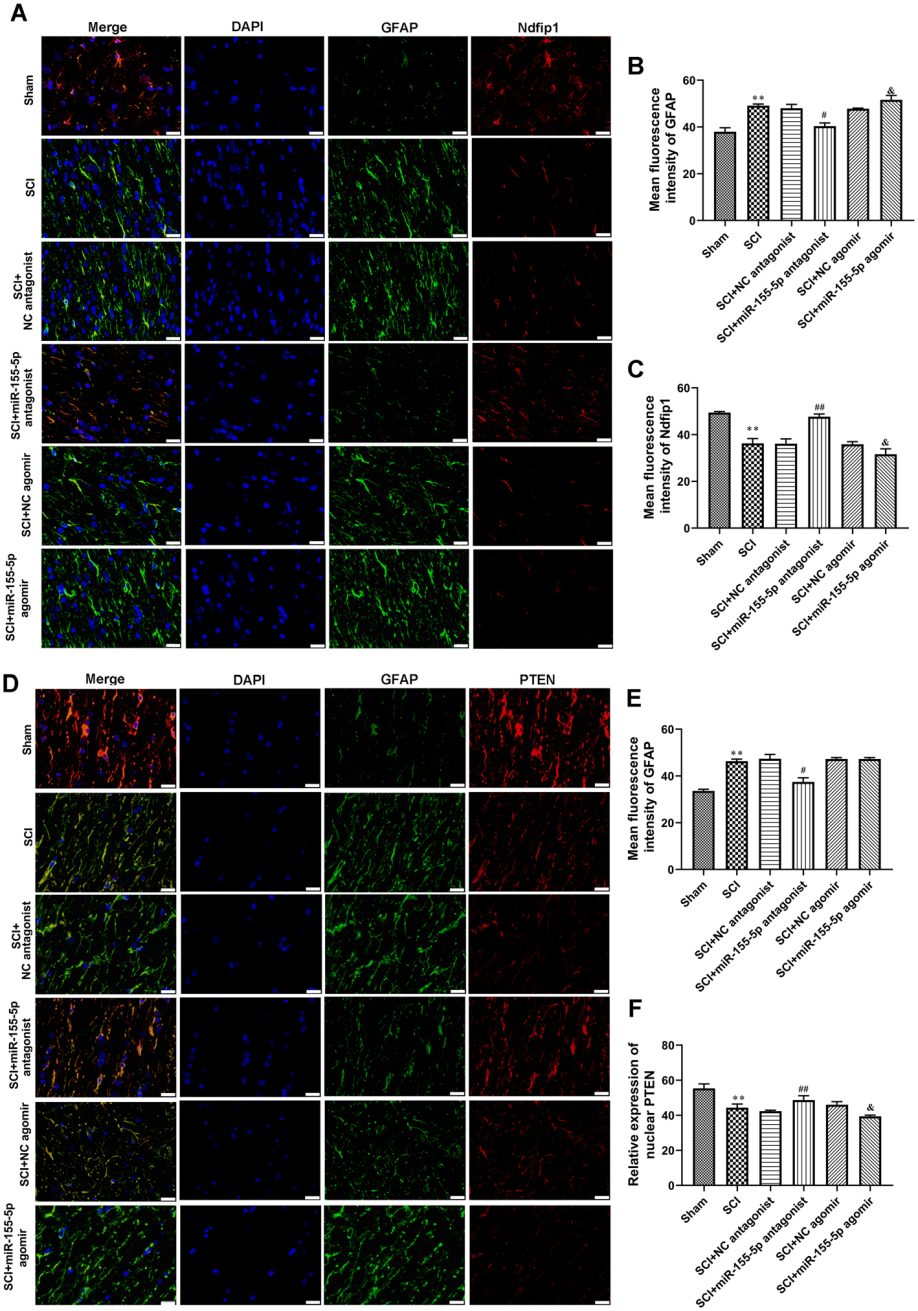
Immunofluorescence stain of Ndfip1 and PTEN expression in vivo. **A**–**C** Double immunofluorescence stain of GFAP together with Ndfip1. Scale bars = 50 μm. Astrocyte was labeled immunofluorescent green for GFAP. **D**-**F** Double immunofluorescence stain of GFAP and PTEN. Scale bars = 50 μm. Astrocyte was labeled immunofluorescent green for GFAP. **P < 0.01 vs. Sham, ^#^*P* < 0.05 vs. SCI + NC antagonist, ^##^*P* < 0.01 vs. SCI + NC antagonist, ^&^*P* < 0.05 vs. SCI + NC agomir

To determine whether miR-155-5p can play a role after SCI in vivo, miR-155-5p antagonist, miR-155-5p agomir, and its negative control (NC) were used to modulate the miR-155-5p expression level in a mouse SCI model. We first confirmed an efficient up or down-regulation of miR-155-5p expression by RT-qPCR (Fig. 5A). BBB score was also recorded at different time points. The scores indicated that the miR-155-5p antagonist promoted functional recovery compared with its NC, whereas the miR-155-5p agomir showed the opposite effect (Fig. 5B). To specify the influence of miR-155-5p on the secretory functions after SCI, RT-qPCR was used to measure NGF and BDNF. Indeed, both neurotrophin nerve growth factor (NGF) and brain-derived neurotrophic factor (BDNF) expression could be induced by treatment with the miR-155-5p antagonist and inhibited by miR-155-5p agomir (Fig. 5C and D). Furthermore, in the SCI model, miR-155-5p antagonist promoted Ndfip1 expression, total PTEN expression, and nuclear PTEN expression in spinal cord tissue of SCI rats (Fig. 5C and D). Meanwhile, the miR-155-5p agomir showed the opposite effect (Fig. 5E, J). In addition, double immunofluorescence of GFAP/Ndfip1 and GFAP/PTEN in the spinal cord showed that miR-155-5p antagonist weakened GFAP expression, enhanced Ndfip1 and PTEN expression in astrocytes of SCI rats and miR-155-5p agomir showed the opposite effect (Fig. 6A–C). These results implied that miR-155-5p inhibition eliminated SCI and astrocyte activation in vivo by regulating Ndfip1expression and PTEN nuclear translocation.

### MiR-155-5p Regulated Glial Scar Formation in the Spinal Cord of SCI Rats

**Fig. 7:**
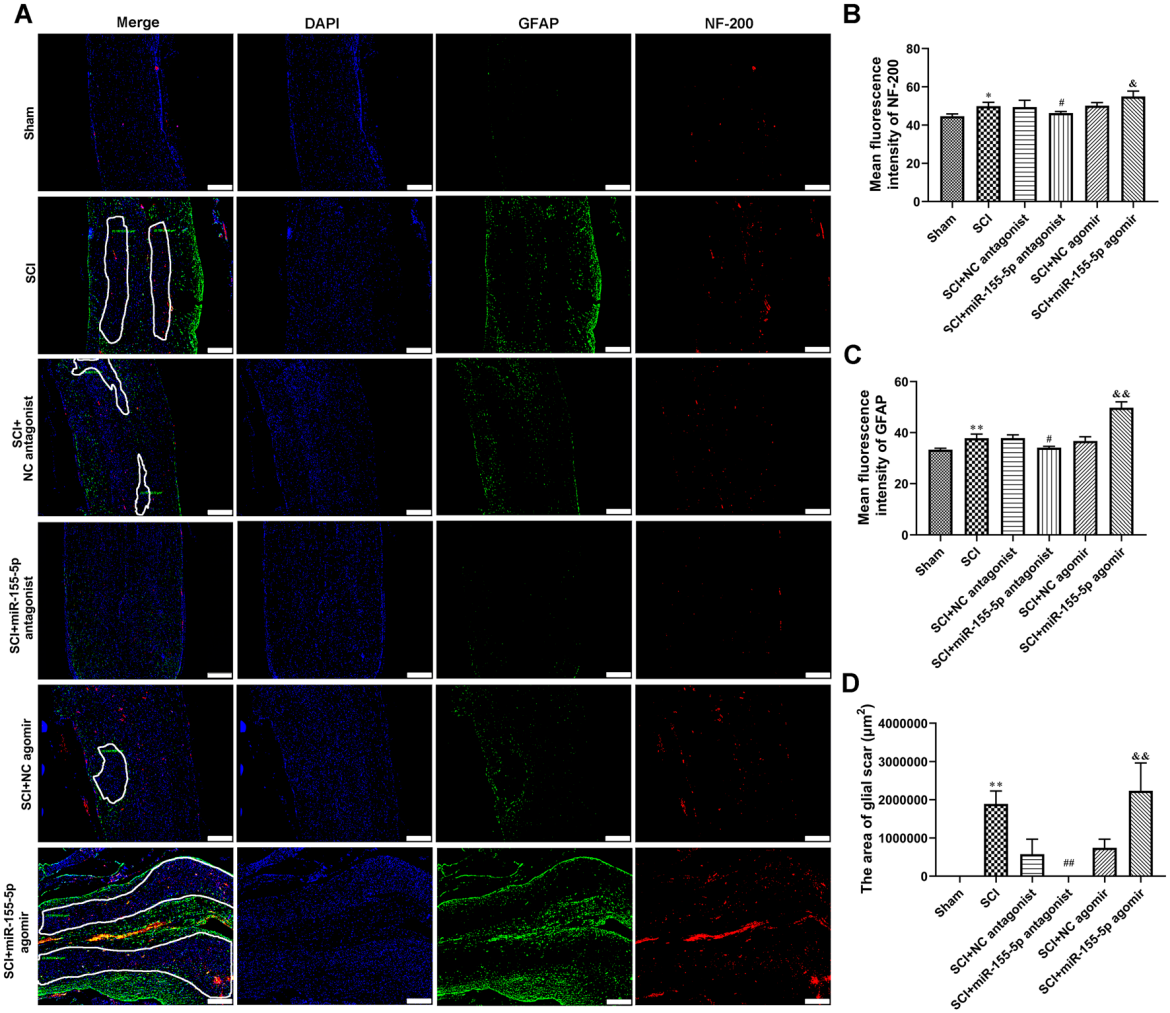
MiR-155-5p regulated glial scar formation in spinal cord of SCI rats. **A–C** Double immunofluorescence stain of GFAP and NF-200. Glial scar formation was marked using white box lines. Scale bars = 50 μm. **D** The area of glial scars was calculate. *P < 0.05 vs. Sham, **P < 0.01 vs. Sham, ^#^*P* < 0.05 vs. SCI + NC antagonist, ^##^*P* < 0.01 vs. SCI + NC antagonist, ^&^*P* < 0.05 vs. SCI + NC agomir, ^&&^*P* < 0.01 vs. SCI + NC agomir

The glial scar formation in the spinal cord was further evaluated by immunofluorescence analysis of GFAP and NF-200. Figure 7A–D showed that compared with the sham group, SCI resulted in significant glial scar formation in the spinal cord. Moreover, treatment of miR-155-5p antagonist decreased the positive levels of GFAP and NF-200, as well as inhibited glial scar formation in the spinal cord of SCI rats(Fig. 7 A–D). The miR-155-5p agomir showed the opposite effect (Fig. 7A–D). The data suggested that miR-155-5p inhibition glial scar formation in the spinal cord of SCI rats.

## Discussion

Reactive astrogliosis is a dominant component of the microenvironment after central nervous system (CNS) injury, which effects may vary depending on the damage extent and injury type [10, 25]. Its crucial involvement in determining the degree of brain pathology and neurological damage, together with its universal participation in tissue response to noxious stimuli, renders astrogliosis an appealing target for therapeutic interventions aimed at promoting nervous tissue preservation and repair [26]. Reactive astrogliosis is characterized by hyperplasia, hypertrophy of cell bodies and cytoplasmic processes, and up-regulation of intermediate filament proteins, such as glial fibrillary acidic protein (GFAP) and vimentin, and ultimately forms a histologically apparent glial scar at the lesion site in the damaged spinal cord [27]. In the early phase after CNS injury, the glial scar plays a key role in sealing the lesion site, restoring homeostasis, preserving spared tissue, and modulating immunity [28]. However, excessive scar formation is one of the major current obstacles to axonal regeneration and functional recovery in the later periods [29]. Many miRNAs were reported to be associated with reactive astrocyte proliferation and the formation of the glial scar after SCI, which might serve as a promising target in the treatment of SCI. MiR-106-3p facilitates functional recovery via inactivating inflammatory microglia and interfering glial scar component deposition after SCI [30]. MiR-21 regulated astrocytic reaction post-acute phase of SCI through modulating TGF-β signaling [19]. MiR-140/BDNF axis regulated human astrocyte (NHA) cell proliferation through PI3K/AKT pathway [31]. MiR − 211/BDNF axis regulated LPS-induced proliferation of normal human astrocyte through PI3K/AKT pathway [32]. In this study, we found that miR-155-5p overexpression promoted astrocyte activation in vitro, as evidenced by the increase of cell proliferation cells and the decrease of apoptosis level. Moreover, miR-155-5p overexpression promoted scar formation in the spinal cord tissue of the SCI rat model. Similarly, Previous research showed that in a model of a spinal contusion injury, miR-155 deletion reduced neuroinflammation and improved locomotor function [21].

Nedd4 family interacting protein 1 (Ndfip1) is an adaptor and activator protein, which can interact with several Nedd4 families to ubiquitinate target proteins [33, 34]. Increasing evidence suggested that Ndfip1 was a neuroprotective protein, and Ndfip1-mediated protein ubiquitination might be a possible survival strategy in neuronal injury [35]. In addition, Ndfip1 could induce PTEN ubiquitination with the cooperative protein Nedd4. Polyubiquitin of PTEN promote its cytoplasmic degradation and monoubiquitin can induce its import into the nucleus [36]. Therefore, it regulates the stability and subcellular localization of PTEN, which may be the main regulatory mechanism of PTEN post-translational modification. A study reported that In vivo, transgene expression of Ndfip1 in the developing brain increased nuclear PTEN and lengthened the cell cycle of neuronal progenitors, resulting in microencephaly [36]. Our data indicated that miR-155 inhibited Ndfip1 expression and PTEN nuclear translocation by targeting 3’-UTR of Ndfip1. Moreover, miR-155-suppressed astrocyte activation was reversed by Ndfip1 overexpression.

It is known that the PTEN gene plays an important role in the recovery of nerve injury. Conditional deletion of PTEN in corticospinal neurons promotes axon sprouting and regeneration after SCI [37]. In a model of SCI, PTEN silencing combined with ChABC-overexpression in adipose-derived stem cells promotes functional recovery [38]. Meanwhile, PTEN silencing increased the regenerative growth of corticospinal tract axons after SCI [39]. MiR-17 targeted PTEN and facilitated glial scar formation after SCI via the PI3K/Akt/mTOR signaling [18]. Neuronal RARβ signaling modulated PTEN activity directly in neurons and via exosome transfer in astrocytes to prevent glial scar formation and induce spinal cord regeneration [40]. In the present study, we found that Ndfip1 overexpression abolished the inhibition effect of miR-155-5p mimic on PTEN nuclear translocation, which may play a role in inhibiting glial scarring.

The shortcoming of this study is the lack of validation experiments that Ndfip1 and PTEN nuclear translocation mediate miR-155-5p regulation of spinal cord injury in the rat model of SCI. In future studies, we will further investigate the role of Ndfip1 overexpression in mediating miR-155-5p-regulated glial scarring in vivo.

In conclusion, our findings demonstrate a novel molecular mechanism by which miR-155-5p regulates SCI injury. These data indicated that Ndfip1 serves as a direct target of miR-155-5p to regulate astrocyte activation, glial scarring, and PTEN nuclear translocation. Thus, miR-155-5p -based therapy has a wide application prospect in SCI.

## Data Availability

The datasets used or analyzed during the current study are available from the corresponding author on reasonable request.
